# Video-assisted thoracoscopic surgery lobectomy for giant intralobar pulmonary sequestration: A case report

**DOI:** 10.1097/MD.0000000000029284

**Published:** 2022-07-22

**Authors:** Yongyong Wu, Zhongrui Ye, Zhongliang He, Xueming He, Xia Hong, Fei Chen, Shunxin Xin

**Affiliations:** Department of Cardiothoracic Surgery, Tongde Hospital of Zhejiang Province, Hangzhou, Zhejiang Province, China.

**Keywords:** intralobar pulmonary sequestration, Video-assisted thoracic surgery

## Abstract

Pulmonary sequestration, which can be divided into 2 main types: intralobar pulmonary sequestration (IPS) and extralobar pulmonary sequestration, is an uncommon congenital condition for which surgical resection is usually indicated. Video-assisted thoracoscopic surgery, as compared with open thoracotomy, has increasingly become the preferred operative procedure in the treatment of PS, owing to less postoperative pain and faster recovery. This report describes a rare and challenging case with a giant IPS undergoing video-assisted thoracic lobectomy.

**Patient concerns:**

A 39-year old woman suffered from recurrent pneumonia for nearly 3 years. An enhanced computed tomography scan performed in our hospital revealed a 12.0 cm × 10.0 cm-sized IPS in the left lower lobe, supplied by an 8-mm aberrant artery originating from the descending thoracic aorta.

**Diagnosis:**

Histology of the resected lobe confirmed the diagnosis of giant intralobar pulmonary sequestration associated with infection.

**Interventions:**

Thoracoscopic left lower lobectomy was performed.

**Outcomes:**

The patient has been discharged from the hospital on the ninth day after surgery with an uneventful recovery, she was in good health after a 1-year follow-up.

**Lessons:**

Although full of challenges, thoracoscopic lobectomy for giant IPS is a safe and feasible surgical procedure associated with reduced surgical trauma and postoperative pain as well as improved cosmetic results compared with traditional thoracotomy.

## 1. Introduction

Intralobar pulmonary sequestration is a rare congenital malformation of the lung, in which an island of the lung parenchyma lacks a normal communication with the tracheobronchial tree and receives arterial blood supply from an aberrant branch of systemic circulation rather than the pulmonary circulation, whereas the venous connection is almost normal via pulmonary veins or systemic circulation. Currently, surgical resection is the best way to treat intralobar pulmonary sequestration (IPS) regardless of symptoms and prevent future complications.^[1–3]^ This paper reports a patient with a long history of respiratory symptoms and contrast-enhanced computerized tomography (CT) scans of the chest confirmed the diagnosis of giant IPS. The patient underwent 2-port thoracoscopic left lower lobectomy.

## 2. Case presentation

**Figure 1.**
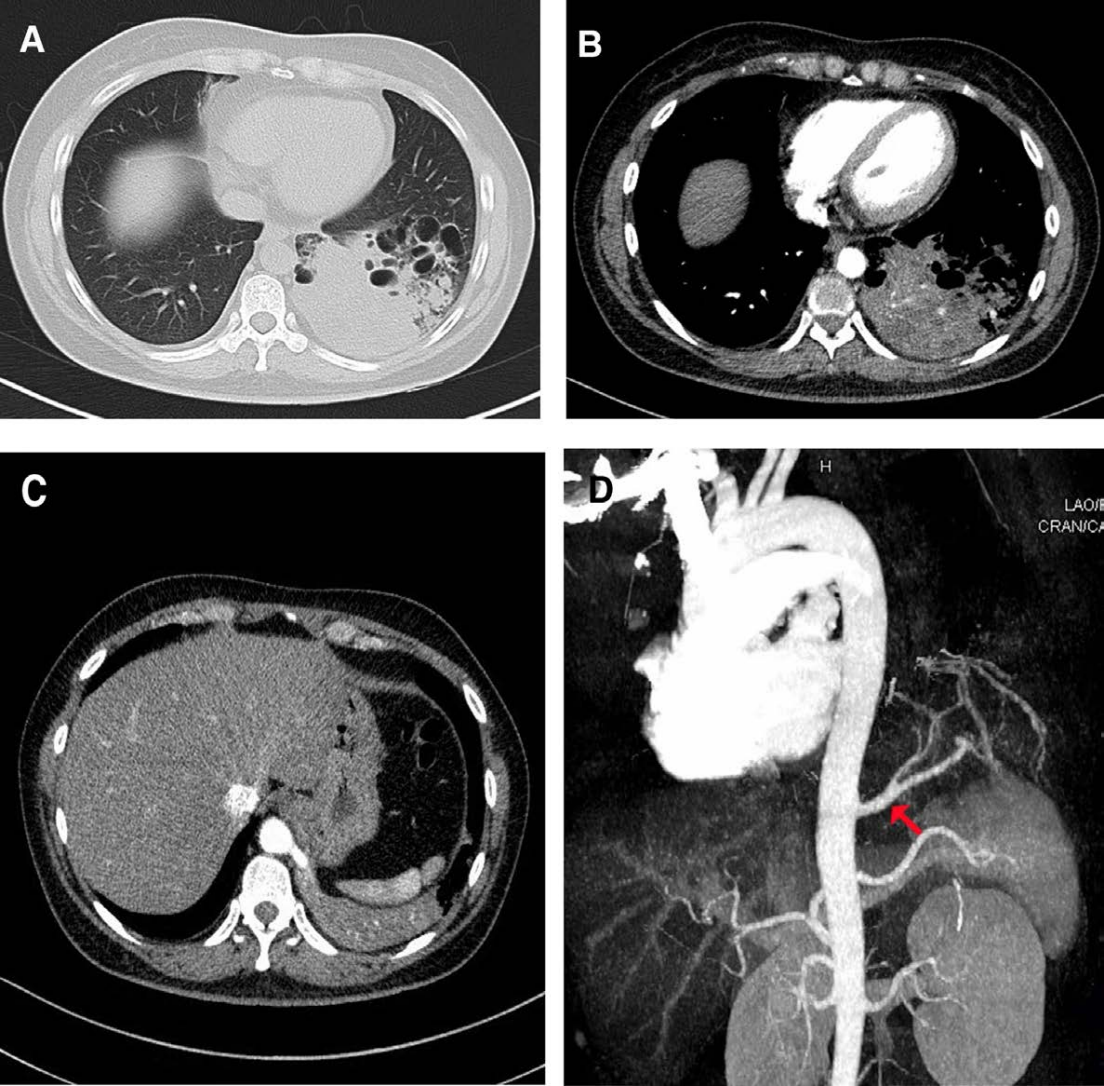
(A and B) Chest contrast-enhanced CT scan of the thorax showed that the left lower lobe had been very extensively replaced by multi-cystic changes and diffuse consolidation of the inflammatory. (C and D) CT scanning 3-dimensional reconstruction confirmed the diagnosis of left lower lobe pulmonary sequestration by clearly demonstrating the aberrant feeding vessels (red arrows).

A 39-year-old female, with a 3-year history of recurrent pneumonia, was admitted to the hospital with a 2-week history of cough with expectoration and fever. A contrast-enhanced chest CT showed a 10.0 cm × 12.0 cm, round mass with multi-cystic changes in the left lower lobe (Fig. 1A and B). It was surrounded by a low-attenuation area that had no apparent connection to the normal tracheobronchial tree. A large aberrant artery measuring 8-millimeter (mm) diameter arose from the descending thoracic aorta and entered the affected lung tissue containing the mass. The systemic artery was more clearly disclosed by subsequent three-dimensional CT angiography (CTA): It was distributed to the segment instead of a normal pulmonary arterial system and was anastomosed with the inferior pulmonary vein (Fig. 1C and D). Fiberoptic bronchoscopy did not reveal any abnormality. There were no abnormalities on pulmonary function tests, arterial gas analyses, and ultrasonographic (US) cardiography. The patient was diagnosed with giant IPS, and 2-port video-assisted thoracoscopic surgery (VATS) left lower lobectomy was scheduled even though it's full of challenges.

**Figure 2.**
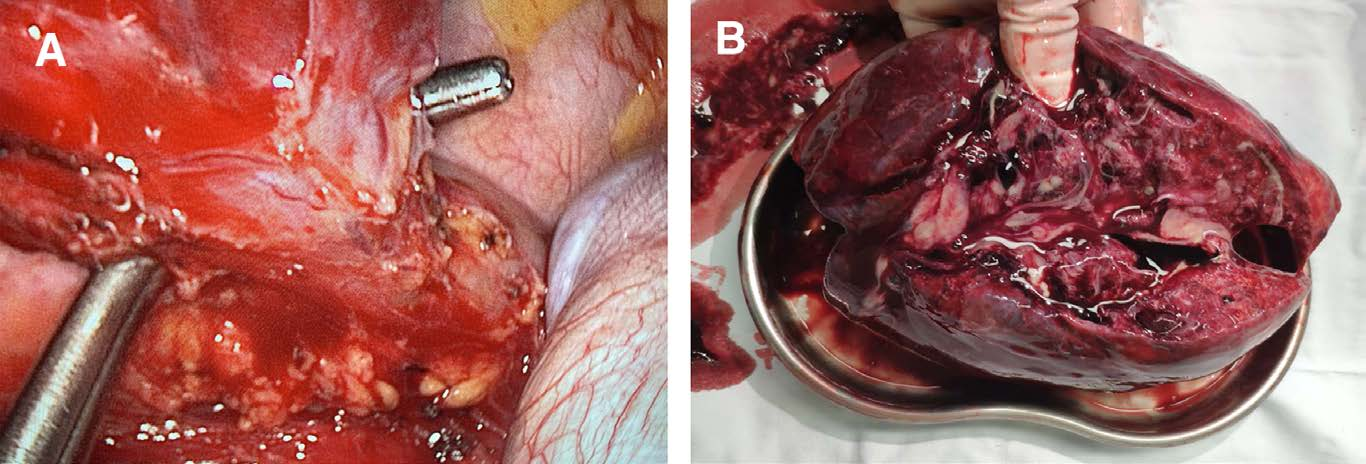
(A) The aberrant artery supplying the giant intralobar pulmonary sequestration was separated. (B) The dissected left lower lobe filled with pus.

Under general anesthesia, a 3.5 cm incision was made in the right 5th intercostal space between the anterior and mid-axillary lines, and a 1.5 cm porthole was made in the 8th intercostal. An Alexis wound protector was applied (Applied Medical, Rancho Santa Margarita, CA). The entire operation was performed using a high-definition 30° 10-mm thoracoscope and a full set of double joint instruments. Intra-operatively, extensive post-inflammatory adhesions were found over the entire posterior-basal aspects of the left lower lobe. Careful release of these adhesions using a combination of energy devices (ultrasonic and electrotome) and long revealed an 8 mm abnormal feeding vessel (Fig. 2A). The larger abnormal feeding vessel was first proximally ligated with a strong silk ligature, then staple-divided using a vascular Endo-GIA stapler (Ethicon Endo-Surgery, Inc). The left lower lobectomy was completed using the standard process, dividing the lobar vein, bronchus, artery, and oblique fissure in order. The specimen was put into a protective bag, and the bag mouth was retracted through the incision. The specimen was removed after it was split by scissors inside the bag (Fig. 2B). The operation was completed in 210 min, and the blood loss was 50 mL. One 24 F chest drainage tube was inserted via the prothole.

The patient's post-operative recovery was without incident, The chest drain tube was removed on the fifth postoperative day and she was discharged on the ninth postoperative day. Histology of the resected lobe confirmed the diagnosis of an intralobar pulmonary sequestration associated with infection.

## 3. Discussion

Pulmonary sequestration is an abnormal segment that lacks communication with the normal tracheobronchial tree and is vascularized by an aberrant systemic artery. Arterial supply of pulmonary sequestration commonly originates from thoracic aorta followed by abdominal aorta and less commonly from intercostal artery, phrenic artery, subclavian artery, and celiac artery. Pulmonary sequestration (PS) has been classified into 2 types: the intralobar type, wherein the segment is located in the normal pulmonary parenchyma without its own pleural covering, and the extralobar type, which is an isolated segment with its own pleural envelope.^[4]^ The clinical manifestations of this disease lack specificity. The severity of symptoms is related to the size of the lung lesions and whether it is co-infected. A few cases have recurrent episodes, and most of them are persistent lung infectious symptoms, such as fever, cough, sputum, hemoptysis, and chest pain. Due to atypical clinical symptoms, PS has a high rate of misdiagnosis and is easily confused with some common respiratory diseases such as lung cysts, lung abscesses, benign lung tumors, and lung cancer.^[5–6]^

Once the disease is discovered, surgical treatment is recommended. The main difficulty of the operation is the management of abnormal blood vessels. Most of the isolated abnormal blood supply in the lung tissue is derived from the descending aorta. The number of abnormal blood vessels is mostly one nourishing blood vessel, there are few reports of 2 or 3 nourishing arteries.^[7–8]^ For patients with suspected IPS, enhanced CT scanning and 3-dimensional reconstruction should be performed, which can clearly show the number and diameter of abnormal blood vessels, it provides great helpfulness for disease diagnosis and intraoperative abnormal blood vessel detection.^[9]^

IPS generally require lobectomy. Pleural adhesions are common troubles in the surgical treatment of IPS, which is mainly related to long-term recurrent infection of diseased lung tissue. Infection can lead to adhesion of the chest near the isolated lung or even throughout the whole pleural cavity. Pleural adhesion was previously considered to be contraindicated for thoracoscopic surgery. However, with the rapid development of thoracoscopic technology, pleural adhesion has been no longer a contraindication of VATS at present, on the contrary, more and more surgeons believe that thoracoscopic surgery has more advantages in the treatment of pleural adhesion than thoracotomy.^[11–13]^ In this case, we chose 2-port thoracoscopic lobectomy and this path is conducive to the disposal of the adhesion between the lower lung and the diaphragm and abnormal systemic circulation supply vessel near the lower pulmonary ligament. The enlarged effect of thoracoscopic surgery on the operative field is conducive to the observation of abnormal vessel at a deeper position, which greatly improves the safety of surgery and reduces the possibility of intraoperative bleeding.

The anomalous blood supply arteries of IPS are mostly located in the lower pulmonary ligament, which lacks a muscle layer, has a thick elastic layer, and from the systemic circulation. The blood vessel wall is hard and fragile. Once it ruptures, bleeding is dangerous. The dissection, separation, and severance of this operation are the difficult and most dangerous parts of the operation. Most scholars advocate treating abnormal arteries before performing lobectomy. However, for severe adhesions in the lower lung ligaments and unclear anatomy, lobectomy can also be performed first, and then dispose of the abnormal blood vessel.^[14–15]^ There is a consensus on the treatment of abnormal blood vessels. Before ligation and severance, they should be free enough to prevent their retraction and bleeding, which may cause serious consequences. In this case, the diameter of the abnormal blood vessel is 8 mm. We used the method of clipping the proximal end of abnormal vessel with first ligated proximally using a non-absorbable suture and then divided using a vascular endo stapler. This method is thought to reduce pressure on abnormal blood vessels in the systemic circulation and reduce the risk of rebleeding during the perioperative period.

In conclusion, the thoracoscopic lobectomy for giant IPS is a feasible and safe approach for the surgeons experienced with VATS lobectomy, and it may provide better cosmetic results with less postoperative pain. Complete preoperative imaging examinations to clarify abnormal arteries, careful dissection and separation of adhesions during the operation, and reasonable treatment of abnormal blood vessels is the key to a success.

## Acknowledgments

We thank the patients and family members for their participation.

## Author contributions

Conceptualization: Yongyong Wu.

Data curation: Zhongliang He.

Formal analysis: Zhongrui Ye.

Methodology: Xia Hong, Fei Chen.

Visualization: Xueming He.

Writing – review & editing: Yongyong Wu, Xinshun Xin.
